# Illness Beliefs in End Stage Renal Disease and Associations with Self-Care Modality Choice

**DOI:** 10.1371/journal.pone.0154299

**Published:** 2016-07-01

**Authors:** Anuradha Jayanti, Philip Foden, Alison Wearden, Sandip Mitra

**Affiliations:** 1 Department of Nephrology, Central Manchester Hospitals NHS Trust, Manchester, United Kingdom; 2 Department of Biostatistics, University of Manchester, Manchester, United Kingdom; 3 School of Psychological Sciences, University of Manchester, Manchester, United Kingdom; University of Utah School of Medicine, UNITED STATES

## Abstract

**Background:**

Interest in self-care haemodialysis (HD) has increased because it improves patients’clinical and quality-of-life outcomes. Patients who undertake self-management for haemodialysis may hold illness beliefs differently to those choosing institutional care at the time of making the modality choice or moulded by their illness and dialysis treatment experience. Illness perceptions amongst predialysis patients and in those undertaking fully-assisted and self-care haemodialysis are being investigated in a combined cross-sectional and longitudinal study.

**Study Design:**

The study data are derived from the BASIC-HHD study, a multicentre observational study on factors influencing home haemodialysis uptake. 535 patients were enrolled into three groups: Predialysis CKD-5 group, prevalent ‘in-centre’ HD and self-care HD groups (93% at home). We explore illness perceptions in the cross-sectional analyses of the three study groups, using the revised Illness Perception Questionnaire (IPQ-R). Predialysis patients’ illness beliefs were reassessed prospectively, typically between 4 and 12 months after dialysis commencement.

**Results:**

Illness belief subscales are significantly different between in-centre and self-care HD groups. In a step-wise hierarchical regression analysis, after adjustment for age, education, marital status, diabetes, dialysis vintage, depression, anxiety scores, and IPQ-R subscales, personal control (p = 0.01) and illness coherence (p = 0.04) are significantly higher in the self-care HD group. In the predialysis group, no significant associations were found between illness representations and modality choices. In prospectively observed predialysis group, scores for personal control, treatment control, timeline cyclical and emotional representations reduced significantly after commencing dialysis and increased significantly for illness coherence.

**Conclusions:**

Illness beliefs differ between hospital and self-care haemodialysis patients. Patient’s affect and neurocognitive ability may have an important role in determining illness beliefs. The impact of modality upon illness representations may also be significant and remains to be explored.

## Introduction

End stage renal disease (ESRD) is a global health concern associated with increased morbidity and mortality[1]. With increasing impetus on self-care in ESRD, home haemodialysis (home HD) has seen resurgence in physician-level and patient-level interest and most recently, in practice[2] with the national uptake of home haemodialysis in the UK, rising to 4.7% in 2013[3]. This increase in uptake is also backed by research which has demonstrated benefits of intensive haemodialysis such as that carried out at home, not limited only to clinical and quality-of-life outcomes, but also to cost-effectiveness[4–13]. Self-care HD affords the patient greater autonomy, but necessitates significant level of engagement not only at the outset, but throughout the course of illness management. Patients, who undertake self-management using complex technology for haemodialysis either in their own homes or in hospitals, may have illness beliefs different to those choosing institutional care. Equally, as illness perceptions are not fixed but shaped by the knowledge and experience of both the illness and its treatment, those who experience self-care haemodialysis may develop a different set of illness beliefs from those who experience centre-based haemodialysis.

According to the Common Sense Model of Self-Regulation (CSM)[14], when patients are confronted with a threat to their health, such as in permanent kidney failure starting dialysis as a life sustaining therapy, they draw on their personal models of that health threat to guide their behavioural and emotional responses to it. These models comprise a set of “cognitive representations” or beliefs about the threat, and a set of “emotional representations” or emotional responses to the threat. Together, cognitive and emotional representations are referred to as illness perceptions; the two sets of representations are held to drive different sets of responses, but to be interdependent, so that beliefs about the health threat impact on emotional responses to the threat, and vice versa[14]. Illness perceptions are personal and may be idiosyncratic, and are derived both from concrete perceptual experiences of illness (e.g. the experience of symptoms) and from abstract sources of knowledge (e.g. information from health care professionals such as predialysis education before start of dialysis, or in the media). According to the CSM, the effectiveness of behavioural responses to cope with the health threat, which may include seeking medical help and self-management behaviours, is continually appraised and the information gained from these appraisals may be used to modify and update illness perceptions[14]. Research using the framework of the CSM has led to the specification of the dimensions of cognitive representation of illness[15, 16].

The illness perceptions of patients with ESRD have received much attention in recent years. It is apparent from a number of studies examining the association between illness perception and outcomes in ESRD patients that personal illness beliefs have a predictive value. Illness perceptions have been shown to be associated with depression, health related quality-of-life, adherence with treatment (fluid and medications), and survival[17–22]. As noted above, illness perceptions are thought to be constantly updated as patients acquire new knowledge and experience of their illness[23]. In a longitudinal study of HD patients, over a 2-year follow-up period, patients had fewer negative emotional reactions to the illness, better understanding of the illness, and improved perception of treatment control[24]. Similarly, illness understanding in dialysis patients varies between patients as a function of the length of time on dialysis over a wide range of durations [25] and in the same patient within the first year on dialysis[26]. In published literature, the impact of home-based dialysis modalities on emotional well-being has been explored in small groups of patients[18]. Information on the extent to which illness perceptions influence adjustment in ESRD especially in relation to the different treatment modalities is largely limited to hospital HD vs home-based dialysis modalities, a significant component of the latter being peritoneal dialysis, another home-based renal replacement therapy. The technological complexity of home HD (particularly during the training phase when patients learn self-cannulation) and the intensive rigorous routine of daily schedules present challenges of a different magnitude to peritoneal dialysis. Therefore, cognitive and emotional representations of patients who undertake self-management in the home HD context is important to understand the disconnect between the clinical benefits and the uptake of the modality.

In the current study, we have explored illness perceptions amongst recipients of hospital and self-care haemodialysis and of those participants in CKD stage-5, predialysis, who have made a modality choice. We have examined whether there are differences in illness perceptions in patients receiving home vs hospital-based haemodialysis. On the basis of the common-sense model, we hypothesize that due to different illness and treatment experiences in the three groups, perceptions will vary as a function of treatment type. More specifically, we hypothesized that as a result of illness experience, self-care haemodialysis patients would have greater illness coherence, personal control and treatment control. Also, as patients acquire information, these beliefs would change significantly with commencement of dialysis, irrespective of modality type. Furthermore, we hypothesised that a higher score on ‘positive’ beliefs about illness (defined as higher illness coherence, personal control and treatment control) was associated with choice of self-care therapy in the predialysis stage.

## Methods

The IPQ-R study data are derived from data ascertained for the BASIC-HHD study[27]. The BASIC-HHD study is a comprehensive and systematic study of barriers and enablers of the uptake and maintenance of home HD therapy. The study involves five UK centres, with variable prevalence rates of home HD. An integrated mixed methodology (convergent, parallel design) has been adopted for the BASIC-HHD study in a combined cross-sectional and prospective study design. The methodological details and scope of data collected in the BASIC-HHD appear in a published protocol[27].

### Participants and Procedure

Data presented here are derived from the cross-sectional and prospective segments of the BASIC-HHD study. 535 patients were enrolled into three groups. Predialysis patients for the CKD-5 group, prevalent ‘in-centre’ HD patients were approached if they fulfilled eligibility criteria and complete study specific questionnaires. All self-care haemodialysis patients (93% at home) from each participating centre were also approached. Predialysis patients were contacted consecutively from the predialysis clinics and hospital haemodialysis patients were contacted in consecutive order across all shifts until the centre target for recruitment was reached. Most participants approached were willing to engage with the study and reasons for declining participation included a lack of interest in research participation, and ‘research’ fatigue. Psychological measures employed in this study were a part of compilation of questionnaires. HD patients returned the questionnaires on the same day or within a couple of dialysis sessions ‘in-centre’. Home HD patients returned it by post, as did the pre-dialysis, CKD-5 patients. Neuropsychometric assessments were carried out ahead of dialysis commencement. Visually impaired patients could respond to IPQ-R questions posed to them by the research team member. A small subset of predialysis patients(n = 42) commenced dialysis and these patients completed the study questionnaires again between months 4 and 12 post dialysis commencement.

### Measures

The Revised Illness Perception Questionnaire (IPQ-R) was used to measure illness representations. The psychometric properties of the IPQ-R have been previously tested on centre-based HD patients, and the structural validity, internal reliability, test-retest reliability, and discriminant validity are within acceptable limits. The IPQ-R assesses nine components of illness representation in three sections. It is a generic instrument, designed to be adapted for use with different health conditions. For the present study the term “my kidney disease” was used to describe the patient’s illness. The first section seeks to establish *Identity* where participates are asked ‘yes/no’ questions about 14 different symptoms and if they believe these symptoms are related to their kidney disease. This aspect has not been considered for analysis in the present study.

The second section contains 38 questions addressing 7 subscales. Subscales which theoretically represent positive beliefs about the controllability of the illness and a personal understanding of the condition include *personal control*, *treatment control*, and *illness coherence* dimensions. High scores on *identity*, *timeline*, *consequences*, and *timeline cyclical* scales demonstrate negative beliefs about the number of symptoms attributed to the illness, the chronicity of the condition, consequences of the illness, and the cyclical nature of the condition, respectively. The third section focusses on ‘causes’ and includes 18 patient-perceived causes of underlying kidney disease (i.e., lifestyle, hereditary, stress, chance, drugs etc.). Sections 2 and 3 require participants to respond using a 5-point Likert scale (strongly disagree to strongly agree). Cronbach’s alpha for all subscales was ascertained as a measure of internal consistency in the study group.

Additionally, all study participants completed a compilation of questionnaires. In order to examine the potential impact of patient’s affect and cognitive ability on illness perceptions, additional instruments analysed in the present study include Beck Depression Inventory II (BDI), State and Trait Anxiety Inventory-Trait (STAI-T) and the modified mini-mental state examination (3MS). The scores from these instruments were considered in ordered categories for analyses: BDI (0–10, 11–15, 16–20, 21–25, 26–30, 31+), STAI-T (20–29, 30–39, 40–49, 50+). 3MS (94–100, 86–93, 81–85, 76–80, ≤75).

### Missing data

**Fig 1:**
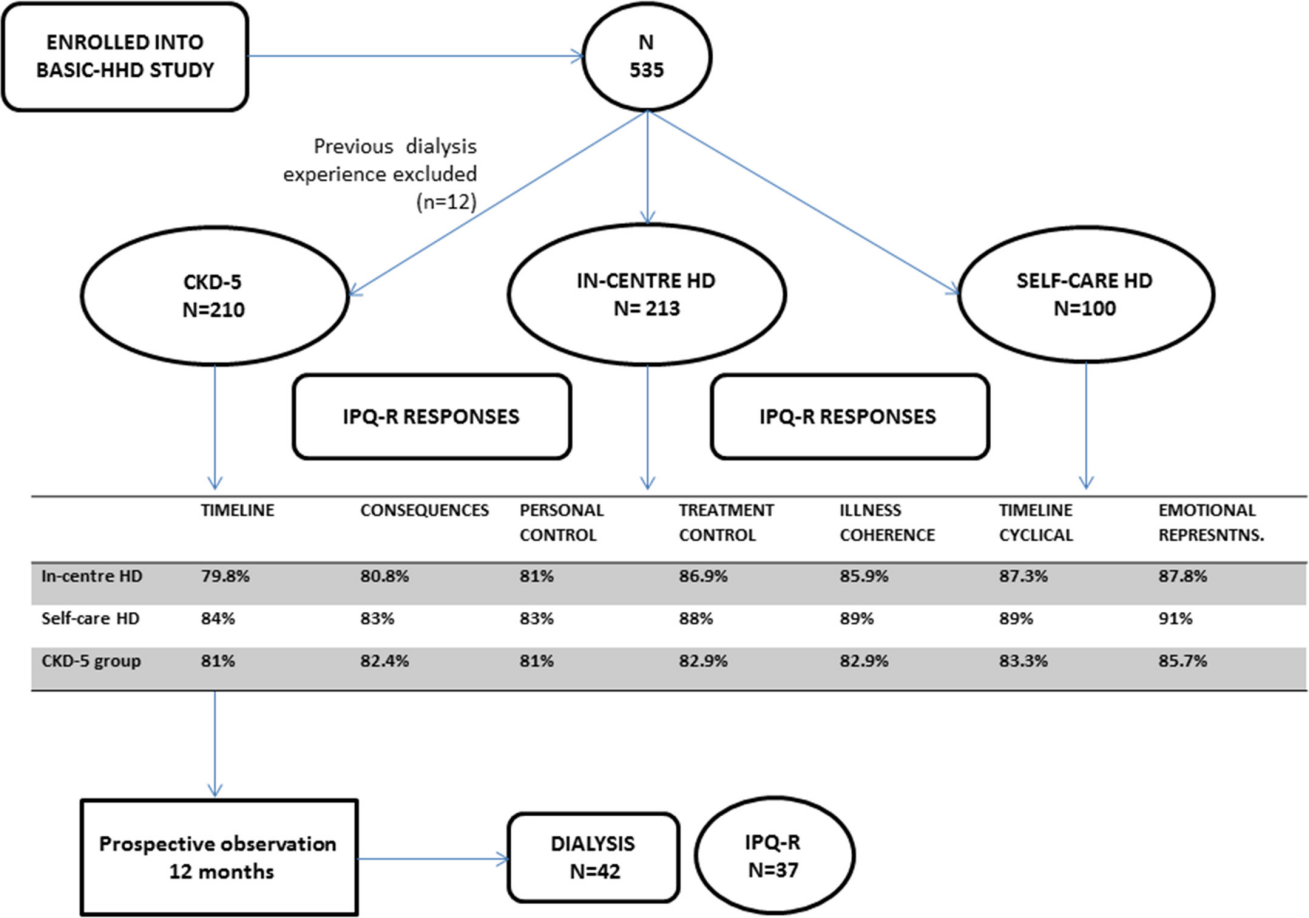
Study flow chart demonstrating patient recruitment and data completeness.

Overall the study had excellent data completion across all instruments used in the study (>82%). The IPQ-R subscales were complete in >80% of the responses from all three study groups across all study subscales (Fig 1).

### Statistical Analyses

Analyses were performed using SPSS 22 and STATA 14. Patient characteristics between groups were assessed using chi-squared tests and Kruskal-Wallis tests. The conventional two-sided 5% significance level was used. Separate analyses were undertaken for the combined haemodialysis group (self-care and hospital) and the predialysis group. Missing data (using the same predictor variables) were analysed using chi-square test, Fischer’s exact test, T-test and Mann-Whitney U test.

For the combined haemodialysis group, a multivariable backwards stepwise regression was performed to ascertain the illness perception variables that have the most significant association with the HD modality. The dependent variable was the modality group. The predictor variables (clinical, demographic, psychological and cognitive tests) which were significant at the 15% significance level in the single variable analysis were included in the multivariable models. Variables were removed, until only those statistically significant at the 5% level remained. Hierarchical regression was also used to assess the significance of differences in illness beliefs between study groups in an adjusted analysis that accounted for clinically important variables. In stage one: age, education level, marital status, dialysis vintage and diabetes status were added. In stage two BDI and STAI-State and Trait were included and at the final stage, the IPQ-R subscales were added. In the predialysis group, multiple regression including the seven subscales was considered with modality choice as the outcome. Any patients in this predialysis, CKD 5 group, who had previous experience of dialysis were removed from the analysis (n = 12). Paired analysis of IPQ-R subscales using the Wilcoxon signed rank test and paired t-test was conducted for the prospective analysis of CKD5 patients who commenced dialysis.

### Study Registration

This study has been reviewed and approved by the Greater Manchester West Health Research Authority National Research Ethics Service (NRES) Reference number: 12/NW/0170. The study is on the NIHR portfolio (ID 12346). Written, informed consent from participants was obtained for the study.

## Results

Recruitment into the study and data completeness (>80%) has been presented in the study flow chart (Fig 1). Numbers of participants in each group include: 210 (predialysis), 213 ‘hospital-based’ haemodialysis and 100 ‘self-care’ haemodialysis patients. Predialysis patients were followed up for 12 months. 42 patients commenced dialysis by the end of this period and information on IPQ-R was available for 37 of them.

### Responders vs non-responders

There are no statistically significant differences in characteristics between those with no missing data and those with some missing data in the HD groups. There is statistically significant difference in the proportion of non-white patients with missing data compared to those with no missing data in the predialysis group. This is likely related to lack of knowledge of English language, sufficient to respond to the questions.

### Patient characteristics in the different study groups

Characteristics of patients included in the study have been presented in Table 1. The three study groups are different from each other with respect to age, education level, dialysis vintage, Charlson comorbidity index, cause of ESRD, peer patient education, presence of informal care-giver and in the overall numbers of patients from each study centre. Between patients in the dialysis groups, the self-care cohort was relatively younger, had higher levels of post-high school education, had lower prevalence of diabetes, often had an informal care-giver and had greater number of dialysis sessions per week compared to the ‘in-centre’ HD recipients. Between groups overall, there was no difference in anxiety and depression scores.

**Table 1:** Characteristics of patients included in the study.

Variable	CKD-5(N = 210)	Hospital HD (N = 213)	Self-care HD (N = 100)	P-value (3-group comparison)
**Age**^1^	62.00 (51.75–69.00)	59.00 (46.50–68.00)	53.00 (44.00–59.75)	<0.001
**Education**^2^ **–**(post-high school)	50/205 (24.4%)	38/203 (18.7%)	42/97 (43.3%)	<0.001
**Ethnicity**^2^ non-white	21/210 (10.0%)	26/212 (12.3%)	13/100 (13.0%)	0.67
**Employment**^2^ Retired	104 (49.5%)	95 (45.0%)	35 (35.4%)	<0.001
Unemployed	40 (19.0%)	74 (35.1%)	25 (25.3%)	
Salaried/self-employed	66 (31.4%)	42 (19.9%)	39 (39.4%)	
**Dialysis vintage**^3^	-	2.72 (1.11–5.23)	3.68 (1.44–7.12)	0.039^4^
**Number of dialysis sessions per week**^3^	-	3.0 (3.0–3.0)	3.5 (3.0–5.0)	<0.001^4^
**CCI**^1^	5.0 (3.8–6.0)	4.00 (3.0–6.0)	4.00 (3.0–5.0)	<0.001
**Diabetes**^2^	72/210 (34.3%)	65/210 (31.0%)	14/99 (14.1%)	0.001
**Heart failure**^2^	12 (5.7%)	11 (5.2%)	4 (4.0%)	0.82
**BDI (score)**^1^	10.0 (5.0–18.0)	11.0 (5.0–20.0)	10.0 (4.0–20.0)	0.59
**STAI-State (score)**^1^	36.0 (26.8–45.3)	34.0 (27.0–45.0)	35.0 (24.0–43.0)	0.45
**STAI-Trait (score)**^1^	39.0 (29.0–47.0)	37.0 (29.0–47.0)	36.0 (28.0–48.0)	0.69
**3MS (score)**^1^	94.0(89.0–98.0)	91.0 (87.0–96.0)	96.0 (89.0–98.0)	0.001
**Caregiver presence**^2^ **/Alone**	51/206 (24.8%)	66/205 (32.2%)	15/97 (15.5%)	0.007
**CKD 5 education evening**^2^	77 (36.7%)	31 (14.6%)	25 (25.0%)	<0.001
**Peer patient education**^2^	94 (44.8%)	59 (27.7%)	39 (39.0%)	0.001
**Cause of ESRD**^2^				<0.001
Hypertensive nephrosclerosis	41 (19.5%)	14 (6.6%)	10 (10.0%)	
Diabetic Nephropathy	55 (26.2%)	48 (22.6%)	11 (11.0%)	
Glomerulonephritis	18 (8.6%)	33 (15.6%)	16 (16.0%)	
Polycystic Kidney Disease	25 (11.9%)	23 (10.8%)	23 (23.0%)	
Renovascular Disease	5 (2.4%)	9 (4.2%)	0 (0%)	
Chronic Pyelonephritis	8 (3.8%)	16 (7.5%)	6 (6.0%)	
Others^5^	37 (17.6%)	39 (18.4%)	16 (16.0%)	
Unknown	21 (10.0%)	30 (14.2%)	18 (18.0%)	

^1^Median and interquartile range presented with p-value from a Kruskal-Wallis test

^2^Number and percentage with p-value from a Pearson chi-squared test

^3^Median and interquartile range presented with p-value from a Mann-Whitney U test

^4^Two-group comparison

^5^Others include- Myeloma, aHUS, bilateral nephrectomy, cardiorenal syndrome, congenital and inherited renal disorders, nephrocalcinosis, obstructive uropathy and tubule-interstitial disease

Pre-dialysis excludes those who previously had dialysis (n = 12)

### Cronbach’s Alpha

The overall measure of internal consistency was good for the IPQ-R subscales although lower for treatment control, and the individual results are as follows: Timeline (α = 0.80); Consequences (α = 0.73); Personal control (α = 0.76); Treatment control (α = 0.63); Illness coherence (α = 0.90); Timeline cyclical (α = 0.80); Emotional representations (α = 0.88).

### Illness beliefs amongst haemodialysis patients

There are differences in illness beliefs between hospital and home haemodialysis patients. The single variable analysis of illness beliefs in the entire HD study cohort suggests that all subscales of the IPQ-R are associated with BDI and STAI-T scores (Table 2). In the single variable analysis with ‘group’ as the outcome variable, several clinical and psycho-socio-demographic factors are associated with belonging in the ‘in-centre’ vs ‘self-care’ group. With respect to subscales of the IPQ-R, significant differences exist between the two HD groups. Self-care haemodialysis patients have greater perceived timeline scores (p = 0.004) and illness consequences (p = 0.037), higher personal control beliefs (p = 0.037) and greater illness coherence (p = 0.001) (S1 Appendix). All variables that were significantly associated with modality group at the 15% level in the single variable analysis were included in multivariable models with ‘group’ as the outcome variable. In this analysis, younger age, post high-school education, non-diabetic status, having a spouse, and greater sense of personal control and of timeline were significantly associated with the self-caring haemodialysis group. Illness coherence was the last variable to be removed from the model (Table 3).

**Table 2:** Single-variable analysis of the subscales of illness beliefs with clinical and psychosocial variables.

	Age^1^	Education^2^	Dialysis Vintage^3^	Diabetes^4^	Heart Failure^5^	Caregiver presence^6^	Peer patient education^7^	Marital status^8^	BDI^9^	STAI-T^10^	Dialysis sessions/ Week^11^	Ethnicity^12^	Gender^13^	Employ-ment^14^	Group^15^
Timeline*	-0.01 (-0.05, 0.03) 0.61	0.10 (-0.03, 0.23) 0.12	-0.01 (-0.02, 0) 0.038	-0.16 (-0.29, -0.03) 0.016	0.11 (-0.14, 0.37) 0.39	-0.05 (-0.18, 0.07) 0.42	0.07 (-0.20, 0.05) 0.25	-0.19 (-0.41, 0.04) -0.12 (-0.36, 0.12) -0.22 (-0.49, 0.06) 0.30	**-0.01 (0.01, 0) 0.085**	**0 (-0.01, 0) 0.27**	-0.11 (-0.19, -0.03) 0.006	-0.37 (-0.54, -0.20) <0.001	-0.01 (-0.13, 0.11) 0.89	-0.03 (-0.17, 0.12) -0.06; (-0.22, 0.09); 0.71	0.15 (0.03, 0.27) 0.012
Consequences*	0.03 (0, 0.06) 0.055	0.03 (-0.07, 0.12) 0.61	0 (-0.01, 0.01) 0.52	-0.05 (-0.15, 0.05) 0.32	0.06 (-0.13, 0.25) 0.53	0.0; (-0.09, 0.11) 0.84	-0.07 (-0.17, 0.03) 0.15	-0.12 (-0.31, 0.06) -0.08; (-0.27, 0.12) -0.25 (-0.47, -0.03) 0.092	**-0.01 (-0.02, -0.01) <0.001**	**-0.01 (-0.02, -0.01) <0.001**	-0.04 (-0.10, 0.02) 0.21	-0.03 (-0.16, 0.11) 0.71	0.01 (-0.09, 0.10) 0.86	0.07 (-0.04, 0.18) 0 (-0.12, 0.12); 0.32	0.09 (0, 0.19); 0.051
**Personal control**	-0.13 (-0.58, 0.32) 0.57	-0.81 (-2.25, 0.64) 0.27	-0.01 (-0.13, 0.12) 0.90	-1.73 (-3.20, -0.25) 0.022	0.06 (-2.72, 2.84) 0.97	0.5; (-0.90, 1.96); 0.46	-1.11 (-2.49, 0.28) 0.12	-1.82 (-4.48, 0.84) -1.81 (-4.64, 1.01) -1.53 (-4.74, 1.68) 0.60	**-0.08 (-0.14, 0.02) 0.012**	**-0.08 (-0.13, -0.02) 0.004**	0.03 (-0.85, 0.91) 0.94	0 (-1.99, 2.00) >0.99	1.3 (0.02, 2.72) 0.046	-2.70 (-4.28, -1.13) -2.30 (-3.97, -0.63); 0.003	-1.43 (-2.76, -0.09) 0.037
**Treatment control**	0.14 (-0.19, 0.47) 0.39	-0.91 (-1.99, 0.18) 0.10	-0.07 (-0.17, 0.02) 0.12	-1.12 (-2.17, -0.06) 0.039	0.84 (-1.33, 3.02) 0.45	0.45 (-0.62, 1.51) 0.41	-0.19 (-1.21, 0.84) 0.72	-0.40 (-2.36, 1.56) -0.32; (-2.41, 1.78); -0.97 (-3.31, 1.38) 0.85	**-0.06 (-0.10, 0.01) 0.014**	**-0.06 (-0.10, -0.02) 0.003**	-1.05 (-1.64, -0.46) 0.001	-1.39 (-2.85, 0.06) 0.060	0.28 (-0.72, 1.28) 0.58	-1.40 (-2.53, -0.27) -2.01; (-3.22, -0.80) 0.004	0.64 (-0.35, 1.63) 0.20
**Illness coherence**	-0.37 (-0.77, 0.04) 0.076	-1.22 (-2.46, 0.02) 0.054	0.18 (0.07, 0.29) 0.002	1.21 (-0.10, 2.51) 0.069	-0.49 (-3.15, 2.18) 0.72	-0.08 (-1.37, 1.20) 0.90	-0.80 (-2.05, 0.45) 0.21	0.42 (-1.96, 2.81) -0.20; (-2.75, 2.35); 1.84 (-1.02, 4.70) 0.29	**-0.06 (-0.12,—.01) 0.024**	**-0.08 (-0.12, -0.03) 0.001**	0.87 (0.16, 1.58) 0.016	0.51 (-1.31, 2.32) 0.59	0.39 (-0.84, 1.62) 0.53	-1.22(-2.62, 0.19)-1.34; (-2.84, 0.16); 0.15	-2.18 (-3.36, -0.99) <0.001
**Timeline cyclical**	-0.27 (-0.59, 0.04) 0.086	0.19 (-0.84, 1.22) 0.72	0 (-0.09, 0.09) 0.99	-0.51 (-1.53, 0.52) 0.33	-0.18 (-2.27, 1.91) 0.87	0.28 (-0.73, 1.28) 0.59	0.52 (-0.46, 1.50) 0.30	-0.63 (-2.50, 1.24) 0.19; (-1.81, 2.19); -0.07 (-2.30, 2.16) 0.46	**0.08 (0.04, 0.12) <0.001**	**0.08 (0.04, 0.11) <0.001**	0.04 (-0.52, 0.61) 0.88	-0.77 (-2.17, 0.63) 0.28	-1.47 (-2.41, -0.53) 0.002	0.12 (-0.98, 1.22) 1.12; (-0.05, 2.29); 0.099	0.10 (-0.85, 1.05) 0.84
**Emotional representations**	-0.65 (-1.14, -0.15) 0.011	0.92 (-0.69, 2.53) 0.26	-0.07 (-0.20, 0.07) 0.33	-0.71 (-2.34, 0.92) 0.39	0.07 (-3.23, 3.37) 0.97	-0.11 (-1.68, 1.47) 0.89	1.45 (-0.08, 2.99) 0.064	0.52 (-2.29, 3.33) 1.84; (-1.20, 4.88); 0.90 (-2.48, 4.28) 0.44	**0.31 (0.26, 0.37) <0.001**	**0.31 (0.26, 0.35) <0.001**	-0.16 (-1.05, 0.72) 0.72	-1.10 (-3.31, 1.11) 0.33	-2.12 (-3.58, -0.66) 0.004	0.27 (-1.43, 1.97) 2.33; (0.50, 4.16); 0.018	0.28 (-1.20, 1.77) 0.71

Data presented as regression coefficients, 95% CIs and p-values

*Transformation used: LN (35—Variable)–for these variables the regression coefficient is in terms of change in LN(35—Variable)

^1^Per ten years

^2^High school compared to post high school

^3^Per year

^4^No diabetes compared to diabetes

^5^No heart failure compared to heart failure

^6^Not alone compared to alone

^7^No peer patient education compared to peer patient education

^8^Married or partnered compared to widowed, single compared to widowed, divorced or separated

compared to widowed

^9^Per unit increase

^10^Per unit increase

^11^Per session

^12^White compared to non-white

^13^Male compared to female

^14^Retired compared to salaried/self-employed, unemployed compared to salaried/self-employed

^15^Hospital compared to home/self-care

**Table 3:** Multivariable analysis depicting odds ratios for predictors of self-care haemodialysis.

Variable	Odds ratio (95% CI)	p-value
**Age** (per ten years)	0.60 (0.46, 0.80)	**<0.001**
**Education**		**0.003**
High school (reference)	1 (-)	
Post high school	2.86 (1.43, 5.72)	
**Diabetes**		**0.008**
No diabetes (reference)	1	
Diabetes	0.30 (0.13, 0.73)	
**Marital status**		**<0.001**
Married or partner	1 (-)	
Single	0.12 (0.05, 0.33)	
Divorced or separated	0.71 (0.25, 2.00)	
Widowed	0.31 (0.06, 1.50)	
**Timeline** (per score increase)	1.08 (1.00, 1.17)	**0.041**
**Personal control** (per score increase)	1.08 (1.01, 1.15)	**0.018**
**Illness coherence** (per score increase)	1.07 (0.99, 1.15)	**0.088**

(Odds Ratios > 1 = Self-care haemodialysis group)

**Fig 2:**
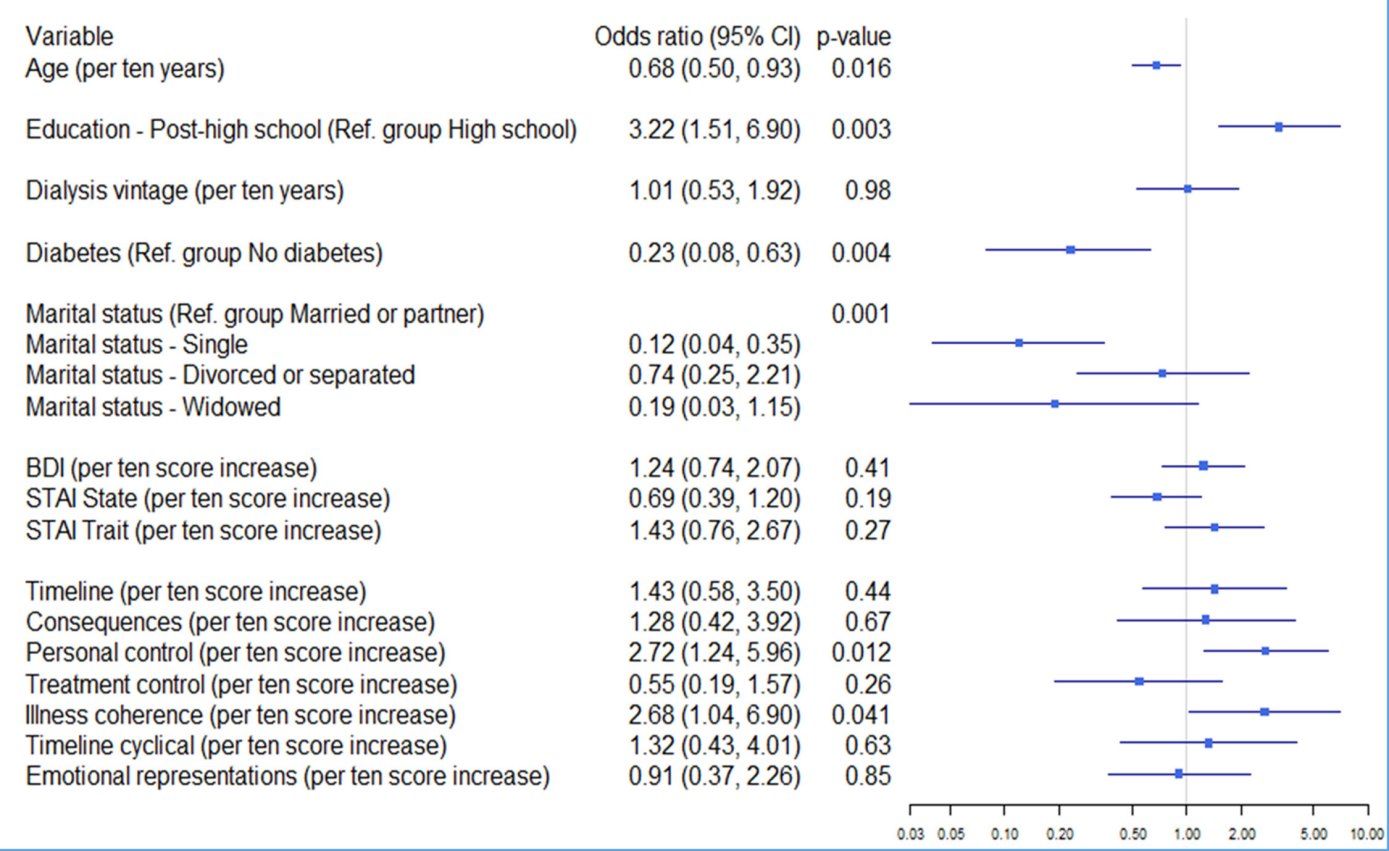
Hierarchical logistic regression to predict self-care vs hospital care group status on the basis of clinical, sociodemographic and psychological factors (N = 214).

We also carried out a step-wise hierarchical logistic regression analysis to predict HD group status (self-care vs in-centre) on the basis of demographic and medical variables, mood and illness perceptions (Fig 2). After adjustment for age, education, marital status, diabetes and dialysis vintage in step 1, BDI and STAI-S/STAI-T score in step 2, the inclusion of all seven illness perception subscales in step 3 shows that the most significant differences between the groups with respect to illness beliefs lie in personal control (p = 0.01) and illness coherence (p = 0.04), higher in the self-care HD group. BDI and STAI, although not significantly different between the two study groups, was found to be correlating with several dimensions of illness perception. Illness coherence is associated with higher 3MS scores in the HD study population.

Personal control or treatment control beliefs held by participants were explored for any significant association with their perceived ability to self-cannulate for haemodialysis. No significant association was determined in a logistic regression analysis at 5% significance level (p = 0.09). However, higher sense of personal control was associated with greater perceived self-cannulation ability.

### IPQ-R in the CKD-5 study group

CKD-5 participants in the cross-sectional cohort made modality choices. We examined the illness beliefs at this stage to see if there is a difference observed between those who choose ‘in-centre’ HD vs home HD and in-centre HD vs the combined home dialysis groups (peritoneal dialysis (PD) and home HD). The results are presented in Table 4. Essentially, no differences were found between the hospital and home HD groups, but, illness coherence was significantly different between choosers of ‘in-centre’ HD and the combined home dialysis group.

**Table 4:** CKD-5 Predialysis Group-Differences in illness perceptions between participants who choose hospital HD vs other modalities (logistic regression analysis).

IPQ-R subscale (per score increase)	Hospital vs Home HD choice		Hospital vs PD+HD combined	
	Odds ratio (95% CI)	p-value	Odds ratio (95% CI)	p-value
**Timeline**	0.92 (0.81, 1.05)	0.21	0.97 (0.88, 1.07)	0.58
**Consequences**	1.07 (0.91, 1.25)	0.43	0.97 (0.87, 1.07)	0.52
**Personal control**	1.09 (0.95, 1.25)	0.20	1.07 (0.98, 1.18)	0.14
**Treatment control**	0.93 (0.77, 1.12)	0.45	0.88 (0.77, 1.01)	0.071
**Illness coherence**	1.11 (0.98, 1.25)	0.11	1.15 (1.04, 1.26)	0.004
**Timeline cyclical**	0.98 (0.82, 1.16)	0.78	0.95 (0.84, 1.07)	0.40
**Emotional representations**	1.03 (0.92, 1.14)	0.66	1.03 (0.95, 1.12)	0.50

The 12 predialysis patients who had non-zero data for dialysis vintage were excluded from the analysis.

OR>1 = Self-care HD choice

HD: Haemodialysis

PD: Peritoneal Dialysis

### IPQ-R and prospective data analysis

In the subset of participants who commenced dialysis (n = 37) in the study period (12 months), their illness perceptions were typically assessed >4 months after commencement of therapy and compared with those obtained at baseline (hospital-based HD (n = 24), home HD (n = 0) and peritoneal dialysis (n = 13) participants). Patients had lower personal control and treatment control 4 months after starting dialysis, perceived their kidney condition to be less cyclical and had less negative emotional representations of their illness. Additionally, participants had greater understanding of their kidney disease as evidenced by higher illness coherence scores after starting dialysis (Table 5).

**Table 5:** Change in illness perception from predialysis to dialysis phase (n = 37).

Variable	Pre-Dial Mean (SD)	Post-Dial Mean (SD)	Change Mean (95% CI)	p-value (paired t-test)
Timeline*	27.0 (22.0–30.0)	29.0 (25.0–30.0)	0.0 (-1.0, 4.0)	0.11^1^
**Consequences**	22.2 (3.5)	21.9 (4.0)	-0.3 (-1.5, 1.0)	0.67
**Personal control**	20.5 (4.1)	18.3 (5.8)	-2.2 (-4.1, -0.3)	**0.027**
**Treatment control**	17.0 (2.8)	15.0 (2.7)	-1.9 (-3.0, -0.9)	**0.001**
**Illness coherence**	17.0 (4.7)	19.9 (5.5)	2.8 (0.7, 5.0)	**0.012**
**Timeline cyclical**	11.2 (3.0)	8.6 (3.4)	-2.6 (-3.8, -1.3)	**<0.001**
**Emotional representations**	18.1 (5.1)	16.1 (5.5)	-2.0 (-3.7, -0.3)	**0.021**

SD: Standard Deviation

*Median and interquartile range due to non-normality (median and IQR change in change column)

^1^Wilcoxon signed-ranks test

### Perceived causes of their illness

**Fig 3:**
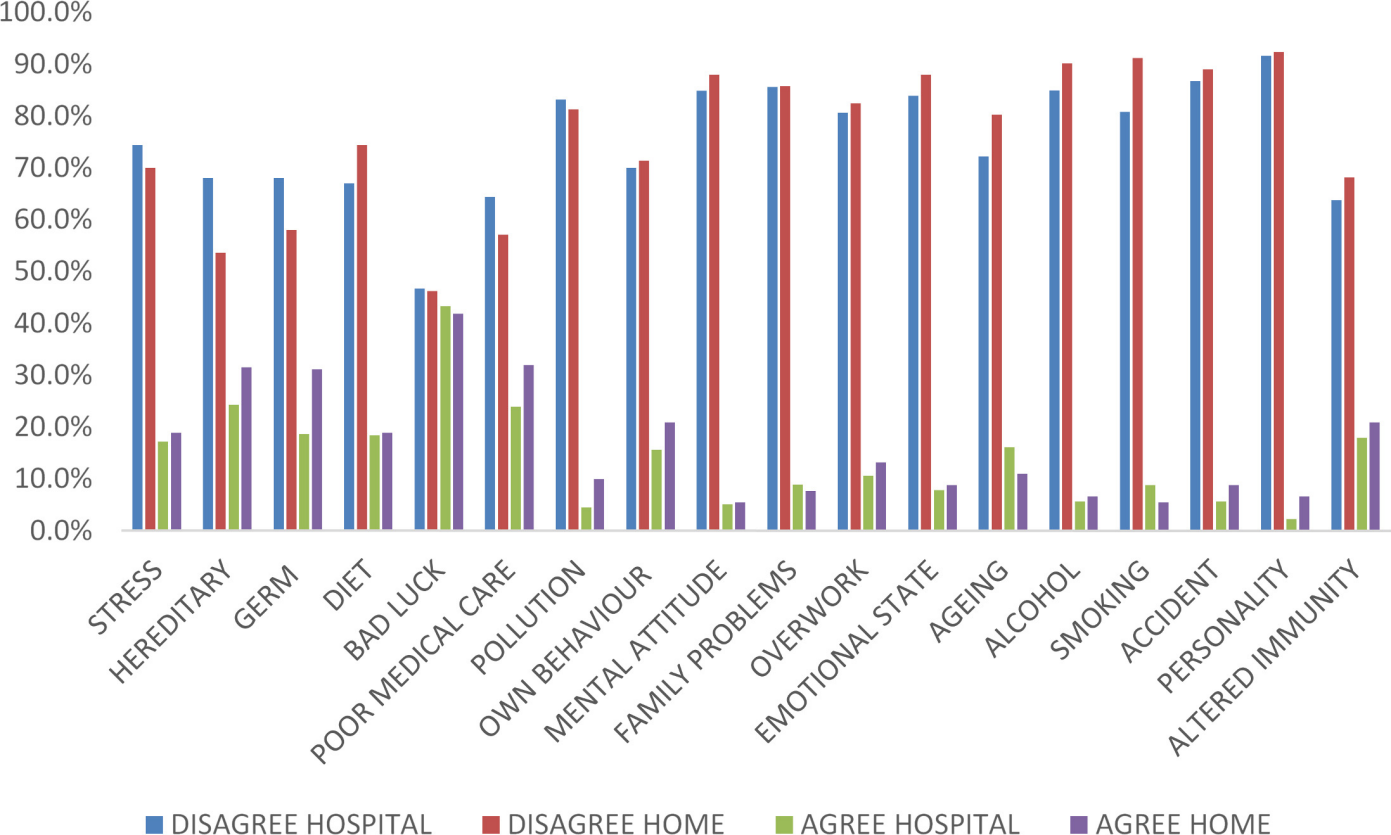
Perceived causes of kidney disease amongst home and hospital haemodialysis patients.

Participants in both groups identified the beliefs they held about the causes of their illness. No significant differences were identified between the groups as to several internal and external causes of their illness, with the exception of smoking (Fig 3). A significantly higher proportion in the self-care group disagreed that smoking was responsible for their kidney disease. Over 40% of participants in both groups attributed their illness to chance or bad luck. A higher proportion of patients in the self-care cohort agree to ‘self-punitive’ factors contributing in some way to the reasons for their illness. About 25% of patients in both groups also attributed ‘poor medical care’ as a cause of their illness.

## Discussion

Beliefs about illness course are potentially important predictors of self-managing behaviour in the chronic illness context, perhaps because they perceive their illness to be serious or severe. Disease duration (timeline) typically refers to whether the disease is believed to be short or long-lasting. In previously published research, patients who viewed their condition (coronary heart disease, asthma, hypertension, diabetes) as chronic were more likely to be adherent with treatment regimens[28–30]. From our data, it is apparent from the multivariable model that self-care haemodialysis patients show greater understanding of the disease chronicity. This perhaps allows individuals to undertake the behaviour that allows self-management–in this context it includes frequent dialysis, fluid and dietary control etc. It is also likely, that the abstract notion of longer illness duration belief (*My disease is chronic…*) is subdued by symptoms stability achieved through better dialysis, in turn promoting self-care haemodialysis. Another construct that is closely aligned with ‘illness course’ belief is the ‘illness consequences’ belief. In the unadjusted single variable analysis, self-care HD patients perceived greater impact of illness consequences upon their lives. This perceived seriousness of their condition possibly leads to taking control of their disease management into their own hands. Equally, it is difficult to discern the perceived effects of home-based HD, the treatment, from disease effects on people’s lives, in the prevalent haemodialysis patients. It is surprising to note that patients with a higher co-morbidity profile in the ‘in-centre’ haemodialysis setting perceive lower illness consequences than their home HD counterparts. This suggests that perception of illness consequences from extra-renal morbidity may temper the expectations from their illness secondary to kidney disease.

In our study, in both the multivariable analysis and hierarchical regression models, perceived ‘personal control’ separated ‘in-centre’ patients from ‘self-care’ haemodialysis patients. As hypothesised, the sense of personal control was significantly greater in the self-care group. It is important to understand if the sense of perceived personal control resulted in self-selection into the self-care HD group or if in fact, this may have been the result of the positive clinical outcomes associated with home HD. The causal direction is difficult to ascertain from a cross-sectional study design. However, we examined a large cohort of predialysis patients who made their modality choices and we examined illness beliefs between groups which made different choices and found no difference in ‘personal control’ beliefs in patients who chose hospital-based HD vs home-based dialysis therapies. In a study by Timmers et al., significant differences in perceptions of personal control and understanding were found between haemodialysis and peritoneal dialysis patients[31], but not so in another study[18]. The reasons for the inconclusive findings could well be the manner in which the different types of PD therapies were considered together for analysis in the studies and the lack of larger patient numbers in the different study groups. This emphasizes the need to look at modality specific clinical and psychological outcomes. The notion of increasing sense of personal control and thereby potentially better psychological adjustment to therapy is an attractive option for patient care[32]. The concepts of shared care and ‘in-centre’ self-care lend themselves to this sense of increased control. This may well facilitate a proportion of patients to consider home haemodialysis in the future, as their illness and treatment understanding improves over time. In fact in our study, perceived ability to self-cannulate was associated with a greater degree of perceived personal control.

Illness coherence was found to be significantly higher in the home HD cohort after adjustment for demographic, clinical and psychological variables. Patients in the predialysis setting are provided education on various modality options. The information they acquire is in the abstract and no difference was observed in our study cohorts between illness understanding in predialysis patients who chose haemodialysis in hospital vs at home. However, this difference is significant between prevalent HD patients in the different locations. In the longitudinal examination of predialysis patients, illness coherence increased significantly after dialysis, likely a result of the actual experience of dialysis. It is however useful to note that illness understanding is significantly different between the hospital HD vs home-based dialysis choosers (combined PD +HD). This is likely a result of a lack of patients choosing home HD compared to PD and also the lack of adjustment for other variables in this subset analysis. This finding suggests that perhaps global cognitive function does play an important role in illness understanding as suggested also by the significant association of this aspect of illness belief with higher 3MS scores in the prevalent HD population in our study. This allows us to explore educational and psychological intervention options in the predialysis phase and beyond to influence the choice of self-care haemodialysis if appropriate.

Beliefs about causes of illness have been studied historically within an attribution theory framework in several clinical conditions. Attribution theory helps classify beliefs into internal and external causative factors. Having a causal theory about one’s illness has been found to be related to better adjustment and coping in some situations. We explored the causal beliefs in HD patients based in hospital and those who self-care. As suggested by authors of the IPQ-R[16], principal components analysis was carried out, but, no satisfactory scales emerged, indicating that different causal beliefs do not cohere. The lack of difference between the two groups with respect to perceived causes of their illness is an important finding. The great majority of patients in both study cohorts, disagreed with the factors posed to them as their causes of illness, although the extent of disagreement was significantly greater in the self-care cohort. Amongst those who agreed, greater proportion of patients in the self-care cohort identified stress and other behavioural factors for their belief in illness causation. Whilst causal beliefs were not significantly different in the overall comparison between the two groups, the positive responses may in fact be a reflection of the day-to-day experience of living with the illness and coping with the treatment regimens at least in some instances (as the original cause of kidney disease may have been diagnosed several years earlier). Therefore, effects of causal attributions on modality choice or adjustment with therapy is best studied prospectively.

This is the first study to report a strong association of objective neurocognitive deficit (3MS) with perceived illness coherence in the context of ESRD. The association between neurocognitive ability, especially, executive functions on self-regulation is well established[33]. However, the systematic examination of the relation between neurobiological factors alongside social-cognitive, emotional, affective and physiological processes in ESRD remains to be explored. That, neurocognitive ability may moderate the association between attitude, intention and behaviour is key to understanding the individual differences in biologically ingrained self-regulatory abilities and the response to health and illness communication. This could be a subject of future research.

In our study, we have explored illness beliefs in large, representative cohorts of hospital and home haemodialysis recipients. The study provides an understanding of illness beliefs prevalent in the hospital vs the self-care haemodialysis groups, and in predialysis vs dialysis participants. It remains to be seen whether interventions may be effective in driving changes in negative illness perceptions amongst predialysis and hospital haemodialysis patients, resulting in a positive impact on patient experience and outcomes in dialysis.We have considered sociodemographic factors and psychological dispositions and neurocognitive function of individual participants in the analyses. The study has had excellent response rates to all study-related questionnaires (82% overall). The internal consistency of IPQ-R in this study cohort is good overall. A key strength of this study is also the way in which some variables have been utilised for data analyses. Strictly dichotomising variables such as BDI or STAI-S/T/3MS, results in loss of information to be ascertained from scores further removed from the cut-off point and as such, the categoriesa have been treated as linear variables.

The cross-sectional design makes causal inferences difficult. The directionality of the significant associations found between self-care HD and patients' perceptions of illness understanding and personal control cannot be established and reciprocal causation cannot be ruled out. Significant proportion of cognitive test scores were missing and could not therefore be considered for the regression models. The duration of prospective observation of predialysis patients did not allow us to capture a large number of dialysis starts.

### Practice implications

Understanding illness beliefs of patients with end stage renal disease is paramount in effecting self-care behaviours. As illness understanding evolves, education and information may be perceived differently in the predialysis and dialysis phases. Designing such education programmes may require more in-depth understanding of patients’ psychological factors. Opportunities to promote self-care should be sought in both these phases of treatment journey. That self-care may impart a greater degree of perceived sense of control and ability is an interesting outcome for patients in any setting- hospital or home-this may influence clinical benefits noted with this treatment modality, reinforcing the message around self-care haemodialysis. The nuances of modality specific illness beliefs are important to comprehend so that interventions may be tailored to individual needs. It would be interesting to further understand if different levels of patient engagement (shared care) in haemodialysis can alter illness beliefs in a positive way so as to influence important clinical and quality-of-life outcomes in hospital HD patients.

In conclusion, illness beliefs differ between hospital and self-care haemodialysis patients. Patient’s affect and neurocognitive ability may have an important role in determining illness beliefs. The impact of modality upon illness representations may also be significant and remains to be explored.

## Supporting Information

S1 Appendix Single variable analysis of study variables with ‘group’ as the outcome variable. (DOCX)
