# Supplementary material for: Illness Beliefs in End Stage Renal Disease and Associations with Self-Care Modality Choice
Source: PLoS One. 2016 Jul 1;11(7):e0154299. doi: 10.1371/journal.pone.0154299 (PMC4930164; doi:10.1371/journal.pone.0154299)
Supplement: S1 Appendix — (DOCX) [file pone.0154299.s001.docx]

S1 Appendix

Single variable analysis of study variables with ‘group’ as the outcome variable

| **Variable** | **Hospital (N=213)** | **Self-care (N=100)** | **P-value** |
| --- | --- | --- | --- |
| Age^1^ | 59.00 (46.50-68.00) | 53.00 (44.00-59.75) | **0.001** |
| Education^2^ –  post-high school | 38/203 (18.7%) | 42/97 (43.3%) | **<0.001** |
| Dialysis vintage^1^ (in years) | 2.72 (1.11-5.23) | 3.68 (1.44-7.12) | **0.039** |
| Diabetes^2^ | 65/210 (31.0%) | 14/99 (14.1%) | **0.002** |
| Heart failure^3^ | 11 (5.2%) | 4 (4.0%) | 0.78 |
| Caregiver presence^2^ –  Alone | 66/205 (32.2%) | 15/97 (15.5%) | **0.002** |
| Opportunity to speak to other patients^2^ | 59 (27.7%) | 39 (39.0%) | **0.044** |
| Marital status^2^ –  Married or partner  Single  Divorced or separated  Widowed | 112 (52.6%)  63 (29.6%)  21 (9.9%)  17 (8.0%) | 71 (71.0%)  13 (13.0%)  13 (13.0%)  3 (3.0%) | **0.002** |
| BDI (score)^1^ | 11.0 (5.0-20.0) | 10.0 (4.0-20.0) | 0.63 |
| STAI State (score)^1^ | 34.0 (27.0-45.0) | 35.0 (24.0-43.0) | 0.57 |
| STAI Trait (score)^1^ | 37.0 (29.0-47.0) | 36.0 (28.0-48.0) | 0.81 |
| Number of dialysis sessions per week^3^ | 3.0 (3.0-3.0) | 3.5 (3.0-5.0) | **<0.001** |
| Ethnicity^2^ –  non-white | 26/212 (12.3%) | 13/100 (13.0%) | 0.85 |
| Gender^2^ –  Female | 75 (35.2%) | 27 (27.0%) | 0.15 |
| Employment^2^ –  Retired  Unemployed  Salaried/self-employed | 95 (45.0%)  74 (35.1%)  42 (19.9%) | 35 (35.4%)  25 (25.3%)  39 (39.4%) | 0.001 |
| Timeline^1^ | 26.0 (24.0-30.0) | 29.0 (26.0-30.0) | **0.004** |
| Consequences^1^ | 24.0 (21.0-27.0) | 25.2 (22.8-27.0) | **0.037** |
| Personal control^4^ | 18.2 (4.8) | 19.7 (5.7) | **0.037** |
| Treatment control^4^ | 15.1 (3.8) | 14.4 (4.1) | 0.20 |
| Illness coherence^1^ | 20.0 (16.0-23.0) | 21.0 (18.9-25.0) | **0.001** |
| Timeline cyclical^4^ | 10.4 (3.8) | 10.3 (3.5) | 0.84 |
| Emotional representations^4^ | 18.2 (5.9) | 18.0 (5.9) | 0.71 |

^1^Median and interquartile range presented with p-value from a Mann-Whitney U test

^2^Number and percentage with p-value from a Pearson chi-squared test

^3^Number and percentage with p-value from a Fisher’s Exact test

^4^Mean and standard deviation with p-value from a t-test
